# Sedative and anxiolytic effects of *Capparis sicula* Duhamel: *in vivo* and *in silico* approaches with phytochemical profiling

**DOI:** 10.3389/fphar.2024.1443173

**Published:** 2024-08-28

**Authors:** Büşra Karpuz Ağören, Esra Küpeli Akkol, Ismail Çelik, Eduardo Sobarzo-Sánchez

**Affiliations:** ^1^ Department of Pharmacognosy, Faculty of Pharmacy, Başkent University, Ankara, Türkiye; ^2^ Department of Pharmacognosy, Faculty of Pharmacy, Gazi University, Ankara, Türkiye; ^3^ Department of Pharmaceutical Chemistry, Faculty of Pharmacy, Erciyes University, Kayseri, Türkiye; ^4^ Instituto de Investigación y Postgrado, Facultad de Ciencias de la Salud, Universidad Central de Chile, Santiago, Chile; ^5^ Department of Organic Chemistry, Faculty of Pharmacy, University of Santiago de Compostela, Santiago, Spain

**Keywords:** *Capparis sicula*, capparaceae, molecular docking, sedative, ethnopharmacology, gaba receptors

## Abstract

The World Health Organization reports that 30% of adults worldwide suffer from insomnia, while 10% of people worldwide suffer with various forms of anxiety. The significant negative effects of conventional medications used to treat anxiety and insomnia, such as abuse, addiction, amnesia, and cognitive and sexual dysfunction, have led to an increased preference for naturally derived substances with fewer side effects. Accordingly, in this study, the sedative and anxiolytic effects of *n*-hexane, ethyl acetate (EtOAc), methanol (MeOH) and water extracts of the aerial parts of *Capparis sicula* Duhamel., which is used for sedative purposes in folk medicine, were evaluated. To evaluate the sedative and anxiolytic effects of each extract, bioassay systems were used including traction and hole-board tests. The MeOH extract of *C. sicula* was the most active extract on *in vivo* traction and hole-board tests compared to Diazepam. From the MeOH extract, major components were isolated, and their structures were identified as three flavonoid glycosides [rutin (1), quercetin-3-*O*-glucoside (2), and quercetin 3-*O*-rhamnoside (3)] using spectral techniques. The most abundant component was determined to be rutin, comprising 8 mg/100 mg dry extract in MeOH extract and 76.7 mg/100 mg dry fraction in fraction C using HPLC. The molecular docking studies evaluated the interaction of isolated flavonoid glycosides with the interaction energies and protein-ligand interaction details of the anxiety-related receptors GABAA and GABAB. For the GABAA receptor, quercetin-3-*O*-glucoside demonstrated the highest docking score. Quercetin-3-*O*-rhamnoside and rutin also show promising interactions, particularly with the GABAB receptor, highlighting their potential as modulators of these receptors. In conclusion, the use of *C. sicula* for sedative purposes in folk medicine has been confirmed for the first time by *in vivo* studies, and its possible active compounds and sedative-anxiolytic mechanism have been determined through phytochemical and *in silico* studies.

## 1 Introduction

Throughout history, plants have been the most important natural resources that people have utilized in almost every aspect of their lives, such as hunting, paying respect for religious rituals, obtaining food and shelter, and solving health problems. Dioscorides, who lived in Anatolia, included detailed information on the use of 500 medicinal plants and medicines prepared from these plants in his 5-volume work “De Materia Medica,” which can be considered as the first pharmacopoeia (Baytop, 1999). The use of plants as medicines started in the early 19^th^ century with Sertürner’s extraction of morphine from opium and the subsequent isolation of compounds such as cocaine, codeine, digitoxin and quinine, in other words, the isolation of bioactive compounds from medicinal plants (Butler, 2004). The use of plants for medicinal purposes is the beginning of the road to modern medicine today.

According to World Health Organization (WHO) research, the number of medicinal plants used for therapeutic purposes worldwide is approximately 20,000. Another WHO data is that approximately 4 billion people world-wide try to solve their health problems using herbal drugs in the first place (Faydaoğlu and Sürücüoğlu, 2011). The three most important factors in the use of medicinal plants are quality, efficacy, and safety, which are similar to those of conventional medicines. In this respect, it is necessary to know the history of medicinal plants used among the people, to record the existing ethnobotanical information before it disappears completely, and to analyze its efficacy and safety in today’s conditions (Leonti and Casu, 2013). Many modern medicines such as taxol, vinblastine, quinine, and artemisinin, are based on traditional medicine and ethnopharmacology (Patwardhan, 2005; Pirintsos et al., 2022). Therefore, it is important scientifically prove the efficacy of plants used in traditional medicine and to identify the compound(s) responsible for the effect.

Among the disorders of the central nervous system (CNS) that are becoming more common are anxiety, sleeplessness, depression, and epilepsy. Based on data from the World Health Organization, about 10% of people worldwide experience anxiety disorders, while 30% of adults have insomnia (Ketcha Wanda et al., 2015). The COVID-19 pandemic in 2020 significantly increased the prevalence of anxiety and depressive illnesses (Institute of Health Metrics and Evaluation, 2023). Anxiety is usually linked to excessive fear associated with a real or unreal stimulus. Phobia-related avoidance and a variety of physical symptoms are frequently present in conjunction with anxiety. Cardiovascular (chest pain, palpitation), neurological (headache, dizziness), respiratory (dyspnea) and gastrointestinal (diarrhea, abdominal pains) symptoms are common physical symptoms of anxiety (Olayiwola et al., 2013). Anxiolytics and sedatives are prescribed to treat and prevent anxiety symptoms, and they usually have a calming effect (Fuentes et al., 2014). In addition to the treatment of anxiety, these agents have clinical uses in the treatment of sleep disorders, for sedation before surgical interventions, for the treatment of epilepsy and seizure conditions, as IV for balanced anesthesia, for the control of ethanol or other sedative withdrawal syndrome, for muscle relaxation in specific neuromuscular disorders, and as diagnostic and therapeutic aids in psychiatry (Katzung et al., 2018). Because of the severe adverse effects of conventional medicines used to treat insomnia and anxiety, such as abuse, addiction, amnesia, cognitive impairment, and sexual dysfunction, there is an affect toward naturally produced compounds with less side effects (Coimbra Diniz et al., 2019).

*Capparis* species belong to the family Capparidaceae and have been traditionally used for centuries. While the first records of their use are found in Sumerians, there are records of their use for medicinal purposes by the Greeks and Romans in later periods (Tlili et al., 2010). In Türkiye, the buds and leaves of *Capparis* species are used as analgesics, diuretics, wound healers, and cell regenerators in the pharmaceutical and cosmetic industries (Bağcı and Şimşek, 1999). *Capparis ovata* Desf. var. *canescens* (Coss.) Heywood is a synonym of *C. sicula* Duhamel known as “delikarpuzu”, grows widely in Mediterranean and Aegean regions of Türkiye. The decoction of the aerial parts of *Capparis sicula* is used as sedative purposes (Tuzlacı and Sadıkoğlu, 2007) and flower buds, fruits and root bark of *C. sicula* are used in traditional medicine for their analgesic, tonic, cell-regeneration, wound-healing, and diuretic properties (Arslan and Bektaş, 2010; Duman and Özcan, 2015).

*Capparis* species have been found to contain numerous phytochemicals such as flavonoids, alkaloids, glucosinolates, furfural derivatives, furoic acid derivatives, 4-hydroxybenzoic acid, vanillic acid, protocatechic acid, succinic acid, 4-vinyl guaiacol, octanoic acid (Yang et al., 2010). In biological activity studies on fruit, flower, root, and root bark extracts of *Capparis* species, it has been reported that they have antioxidant, anti-atherosclerosis (Ness, 2015), antihypertensive, anti-inflammatory (El Azhary et al., 2017), analgesic, anti-hyperlipidemic, hepatoprotective (Aghel et al., 2010), antibacterial, and antifungal activities (Chahlia, 2009; Duman et al., 2013). It is recorded in the literature that these activities of the caper plant may be due to the phenolics, alkaloids (Rathee et al., 2011), terpenoids (Calis et al., 2002), vitamins, and minerals in its composition (Duman et al., 2013; Gull et al., 2015).

*Capparis* species are considered a potential therapeutic agent in the treatment of nervous system and neurodegenerative diseases thanks to the numerous bioactive compounds in their structure. Stigmast-5,22-diene-3β-ol myristate compound isolated from *C. ovata* has been proven to be effective on multiple sclerosis (Ozgun-Acar et al., 2017). In a study conducted by Nazıroğlu et al., it was reported that an ethanol extract prepared from the flowers of *C. ovata* modulated brain oxidative toxicity and epilepsy seizures induced by pentylenetetrazole (Nazıroğlu et al., 2013). In another studies an ethanol extract of the aerial parts of *Capparis decidua* containing flowers and fruits was found to have good sedative and anticonvulsant activity in Wistar albino rats (Benehelah et al., 2000; Goyal et al., 2009).

According to studies, it has been revealed that three basic neurotransmitter systems, such as the gamma-aminobutyric acid (GABA)-benzodiazepine ionophore complex, noradrenergic system, and serotonergic system, have important roles in the formation and maintenance of pathological anxiety (Uzbay, 2002). Studies on anxiety have focused on abnormalities of GABAA receptor function, and the majority of anxiolytics target this GABA complex. A sedative-anxiolytic effect is expected by increasing GABAA neurotransmission (Durant et al., 2010). GABAB receptors show inhibitory effects by causing slow and long-lasting hyperpolarization from GABAA receptors (Yılmaz et al., 2010). The role of GABAB receptors in central nervous system disorders has not yet been definitively determined, but their high levels of expression in the limbic system have been reported to be involved in the regulation of emotinal behaviors (Yazıcı, 2010; Sinclair and Nutt, 2012).

Consequently, this study aimed to evaluate the sedative and anxiolytic effects of the aerial parts of *C. sicula*, which is used for sedative purposes in folk medicine, by bioassay systems, the isolation and quantification of potentially effective compounds, and the determination of the sedative-anxiolytic mechanism of these compounds by *in silico* study.

## 2 Materials and methods

### 2.1 Plant material

The aerial parts of *C. sicula* were collected from roadsides in Beypazarı-Nallıhan road, Ankara. Fresh branches, parts containing flowers, buds and fruits were preferred instead of woody branches. The plant was identified by Prof. Dr. Hayri Duman. A voucher specimen of the plant has been kept in the Herbarium of the Faculty of Pharmacy, Gazi University (Herbarium Number: GUEF3926).

### 2.2 Extraction procedure

The powdered plant material (500 g) was extracted with *n*-hexane, ethyl acetate (EtOAc), and methanol (MeOH) for 5 days, subsequently. The extracted materials were dried by evaporating in a rotavapor at 40°C under low pressure. In addition, an aqueous extract of the powdered plant material was prepared, considering its use in the folk medicine. The plant material (100 g) was dried and powdered, and 2 L of water at 40°C was added and kept in a water bath at 40°C for 1 h. At the end of the period, the extract was filtered, and the filtered extracts were frozen and lyophilized. The yields of the extracts were found to be 0.63%, 1.42%, 22.97% and 20.94% for *n*-hexane, EtOAc, MeOH and aqueous extracts, respectively.

### 2.3 Biological activity studies

#### 2.3.1 Animals

BALB C male mice weighing 20–25 g obtained from the Experimental Animal Production and Research Laboratory (KOBAY) were used in the experiments. Animals were kept in laboratory conditions for at least 3 days before the experiment to adapt to the environment. During the waiting period, the animals were fed with standard pellet feed and water and housed in a laboratory with a room temperature of 21°C–24°C, 40%–45% humidity, 12 h of light, and 12 h of darkness. The experiment was conducted on six groups including the control, *n*-hexane, EtOAc, MeOH, water extracts and the reference groups. Six animals were used in each group in the experiments. The Kobay Experimental Animal Ethics Committee gave its approval to the experiment (Kobay Ethical Council Project Number: 479).

#### 2.3.2 Preparation of test samples

The extracts were suspended in 0.5% sodium carboxymethyl cellulose (CMC) solution with the help of an ultrasonic bath when necessary and administered orally by a special gastric gavage at a dose of 100 mg/kg. The control group animals were given only 0.5% CMC used in the preparation of the test samples. Diazepam, used as a reference material, was administered to mice intraperitoneally at a dose of 1 mg/kg.

#### 2.3.3 Traction test

The method developed by Courvoisier, Laroche and Rousselet was applied to mice (Courvoisier et al., 1957; Laroche and Rousselet, 1990). One hour after the administration of the extracts and diazepam, which was used as a reference substance, the mice were suspended from a horizontally stretched rope which is 40 cm above the tabletop by their forelimbs. The traction times of the hind legs of the mice were recorded with a chronometer and the results obtained from the extracts were compared with the control and reference groups. It was considered normal for a mouse hanging on the rope to pull its hind legs, and a mouse that failed to pull its hind legs to reach the rope was considered to be under sedative effect. The behavior of the animals was also recorded throughout the experimental period.

#### 2.3.4 Hole-board test

The method developed by Clark et al. and File and Wardill was applied to mice (Clark et al., 1971; File and Wardill, 1975). One hour after the administration of the extracts and diazepam, which was used as a reference substance, mice were placed in the center of a 40 × 40 × 25 apparatus with 16 holes with a diameter of 2.2 cm on the floor. The number of times the animals poked their heads into the holes on the apparatus was recorded manually for 5 min.

#### 2.3.5 Statistical analysis of data

The GraphPad Prism 6.0 (San Diego, CA, United States) program was used for statistical analysis. Every parameter was subjected to one-way ANOVA with Dunnett’s Post-Hoc Test. The experimental results’ statistical significance was compared with the control and reference groups was expressed in the following figures:

*: *p* < 0,05; **: *p* < 0,01; ***: *p* < 0,001.

### 2.4 Isolation of the compounds by chromatographic methods

Since the MeOH extract of *C. sicula* showed sedative-anxiolytic activity in all experimental models tested in mice, it was decided to conduct isolation studies on the MeOH extract. The MeOH extract (12 g) was subjected to polyamide column chromatography and eluted with H2O:MeOH with a gradient polarity (100:0 to 0:100). The obtained fractions were combined into four similar groups (Fr. A, Fr. B, Fr. C and Fr. D) by TLC control. The activities of the combined fractions were tested in *in vivo* anxiety experimental models. Since Fraction C was found to be the most active fraction, the isolation study was continued with this fraction. Fr. C was further subjected to chromatographic separation on the reverse phase (RP-18) silica column and eluted with methanol. Compound 1 (93 mg) was obtained as pure yellow powder from Fr. C. Fr. D showed mild activity and so was subjected to a Sephadex LH 20 column, eluted with MeOH. Compound 2 (12 mg) and Compound 3 (9 mg) were isolated from Fr. D in pure forms.

### 2.5 Structure elucidation of the compounds 1–3

The structures of the isolated compounds from the MeOH extract were determined by spectral techniques such as ESIMS (-), 1D- and 2D-NMR (^1^H-, ^13^C-NMR, Agilent,). The chemical names of compounds 1–3 were as follows (Figure 3) rutin (compound 1) (Gyeltshen et al., 2022), quercetin-3-O-glucoside (compound 2) (Zhang et al., 2014), quercetin 3-O-rhamnoside (compound 3) (Gyeltshen et al., 2022). The NMR data of compounds 1, 2 and 3 were given as:

Compound 1: Yellow solid. ESIMS (-) m/z: 609.1462 [M-H]-. ^1^H NMR (600 MHz, Methanol-d4) 7.66 (d, J = 2.2 Hz, 1H), 7.62 (d, J = 8.5 Hz, 1H), 6.86 (d, J = 8.4 Hz, 1H), 6.39 (s, 1H), 6.20 (d, J = 1.9 Hz, 1H), 5.09 (d, J = 7.7 Hz, 1H), 4.50 (s, 1H), 3,79 (dd, J = 10.7 Hz, 1.9 Hz, 1H), 3.61–3.35 (m, 6H), 3.30–3.23 (m, 3H), 1.11 (d, J = 6.2 Hz, 3H). ^13^C NMR (150 MHz, Methanol-d4) δ 178.00, 164.60, 161.56, 157.92, 157.09, 148.39, 144.42, 134.20, 122.12, 121.69, 116.26, 114.63, 104.21, 103.29, 100.99, 98.52, 93.43, 76.76, 75.80, 74.29, 72.50, 70.81, 70.67, 69.96, 68.27, 67.12, 16.45 ppm.

Compound 2: Yellow solid. ESIMS (-) m/z: 463,0891 [M-H]-. ^1^H NMR (600 MHz, Methanol-d4) δ 7.69 (d, J = 2.2 Hz, 1H), 7.57 (dd, J = 8.5, 2.2 Hz, 1H), 6.85 (d, J = 8.5 Hz, 1H), 6.37 (d, J = 2.1 Hz, 1H), 6.18 (d, J = 2.0 Hz, 1H), 5.23 (d, J = 7.6 Hz, 1H), 3.70 (dd, J = 11.9, 2.4 Hz, 1H), 3.56 (dd, J = 11.8, 5.4 Hz, 1H), 3.47 (dd, J = 9.2, 7.7 Hz, 1H), 3,41 (t, J = 9.0 Hz, 1H), 3.33 (t, J = 9.2 Hz, 1H), 3.21 (ddd, J = 9.8, 5.4, 2.4 Hz, 1H). ^13^C NMR (150 MHz, Methanol-d4) δ 178.05, 164.73, 161.61, 157.57, 157.05, 148.42, 144.48, 134.19, 121.76, 121.64, 116.12, 114.57, 104.22, 102.90, 98.50, 93.30, 76.95, 76.69, 74.29, 69.79, 61.12 ppm.

Compound 3: Yellow solid. ESIMS (-) m/z: 447,0953 [M-H]- ^1^H NMR (600 MHz, Methanol-d4) δ 7.32 (d, J = 2.1 Hz, 1H), 7.29 (dd, J = 8.4, 2.2 Hz, 1H), 6.89 (d, J = 8.3 Hz, 1H), 6.35 (d, J = 2.1 Hz, 1H), 6.18 (d, J = 1.9 Hz, 1H), 5.30 (s, 1H), 3.84–3.78 (m, 1H), 3.58–3.51 (m, 1H), 3.49–3.43 (m, 7H), 3.41 (t, J = 9.0 Hz, 1H), 0.94 (s, 3H). ^13^C NMR (151 MHz, Methanol-d4) δ 178.07, 164.68, 161.64, 157.58, 157.06, 148.42, 144.49, 134.18, 121.75, 121.65, 116.33, 116.11, 104.25, 102.86, 98.47, 93.28, 73.66, 71.91, 71.83, 71.74, 16.23 ppm.

### 2.6 Quantification of rutin using the RP-HPLC-DAD method

The quantitative analysis of rutin in the MeOH extract was performed using the RP-HPLC-DAD method. After dissolving the MeOH extract in 25% (v/v) acetonitrile solution to a concentration of 5 mg/mL, membrane filters were used to filter the mixture before it was transferred into vials. Rutin was also prepared in a 25% acetonitrile solution at different concentrations. The HP Agilent 1,260 series LC System, HP Agilent 1,260 4 (quaternary pump) LC Pump and ACE 5 C18 (5 μm, 150 mm × 4.6 mm) column were used. The temperature of the column was 25°C. The mobile phase was a mixture of Solvent A (acetonitrile: formic acid [100:0.1]) and Solvent B (H2O: formic acid [100:0.1]). The peaks were separated using the gradient method with a flow rate of 1 mL/min. After injecting 20 μL of the extract and the standard solutions into the column the chromatograms were recorded from 280 to 330 nm. The following was the flow program: 0 min 8% A, to 25 min 25% A, to 30 min 30% A, to 40 min 56% A, to 42 min 8% A. Finally, the calibration equation and correlation coefficient determined for rutin were found.

#### 2.6.1 Validation

For the quantitative analysis, the external standard approach was used. The standard solution of rutin was prepared with 6 different concentrations 1 ppm to 1,000 ppm. The average of the areas under the peaks for each concentration was determined after standard substances were examined three times in HPLC to generate the calibration curve. The extract was produced at 5 mg/mL concentration and filtered with 0.45 µM membrane filters before injection. The International Conference on Harmonization (ICH) validation and analytical methods Q2 provided the basis for determining the validation parameters (ICH (International Council for Harmonisation of Technical Requirements for Pharmaceuticals for Human Use), 2022). The approach was used to determine the test range, limit of quantitation (LOQ), recovery, and limit of detection (LOD).

### 2.7 Molecular docking interaction of compounds 1-3 with GABA receptors

Molecular docking studies were conducted using Glide XP, a reliable and widely used docking software (Celik and Tallei, 2022; Khairy et al., 2023) to investigate the interaction between anxiety-related receptors and compounds of interest. The target proteins selected for the study were gamma-aminobutyric acid type A (GABAA) receptor (PDB ID: 6X3X) (Kim et al., 2020) and gamma-aminobutyric acid type B (GABAB) receptor (PDB ID: 6UO8) (Shaye et al., 2020). The compounds analyzed included rutin (1), quercetin-3-O-glucoside (2), and quercetin 3-O-rhamnoside (3). The chemical structures and PubChem IDs of the compounds were obtained from the PubChem database. The protein structures were prepared by removing any water molecules and co-crystal ligands from the original PDB files using the Protein Preparation Workflow in Schrödinger Suite (Release 2022-3). The protein structures were optimized and energetically minimized using OPLS4 force fields in Schrödinger Suite, to ensure a stable and reliable docking environment. The compounds of interest were pre-pared using the LigPrep module in Schrödinger Suite. This module generated the 3D conformations and protonation states of the compounds at physiological pH. The docking grids according to co-crystal ligand coordinate were generated using the Receptor Grid Generation tool in Schrödinger Suite. The grids were defined to encompass the active site regions of the target proteins, allowing for the exploration of potential binding sites. For each protein-ligand docking experiment, the compounds were docked into the respective target protein using the Glide XP docking algorithm, The docking was per-formed using default parameters, and the XP (extra precision) scoring function was employed to evaluate the binding affinities of the compounds. The resulting docking poses were analyzed to determine the interaction energies and specific protein-ligand interactions, including hydrogen bonds, π-π stacking, and hydrophobic contacts. The docking scores were calculated in kcal/mol, providing a quantitative measure of the binding affinity between the compounds and the target proteins.

## 3 Results

In this study, the sedative-anxiolytic effects of the aerial parts of *C. sicula* were evaluated. The extract of the aerial parts of *C. sicula* was tested in bioassay systems; traction and hole-board tests were used for sedative-anxiolytic effects.

**FIGURE 1:**
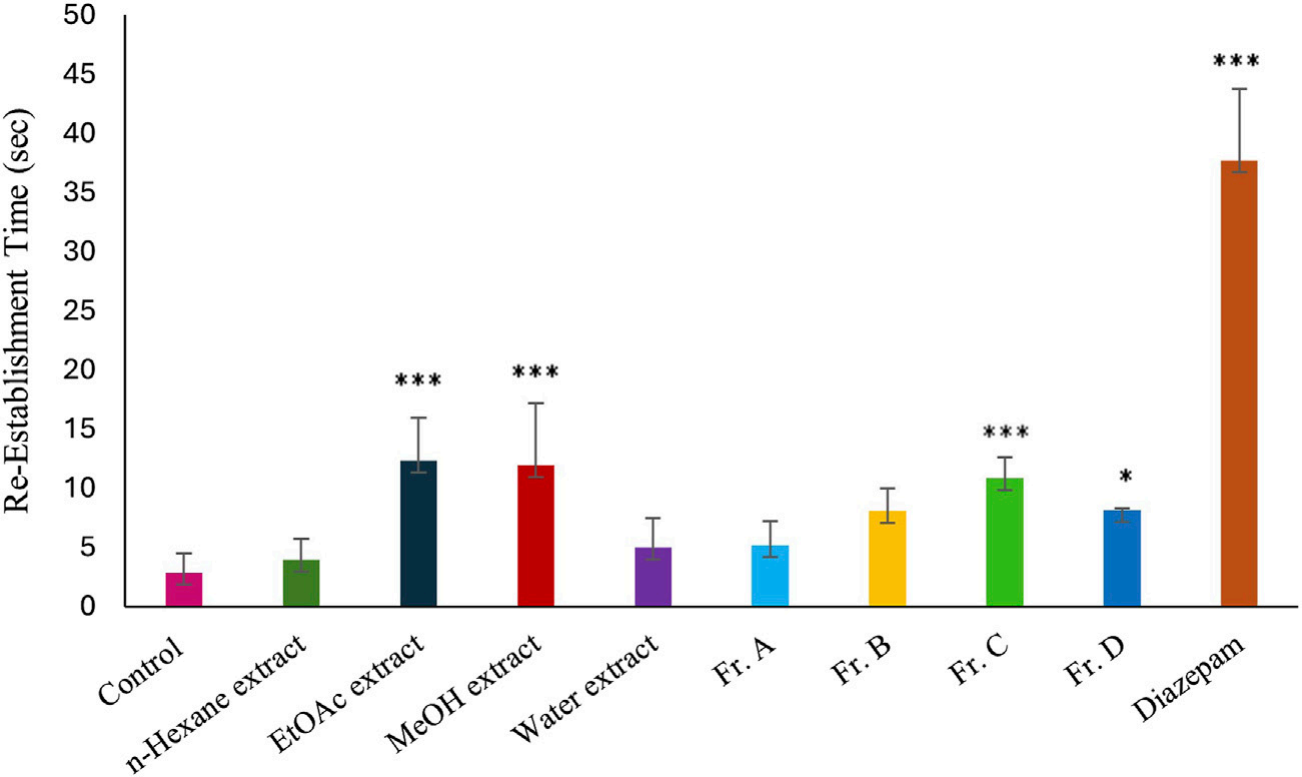
Effects of the test materials on traction test (*: *p* < 0,05; **: *p* < 0,01; ***: *p* < 0,001).

**FIGURE 2:**
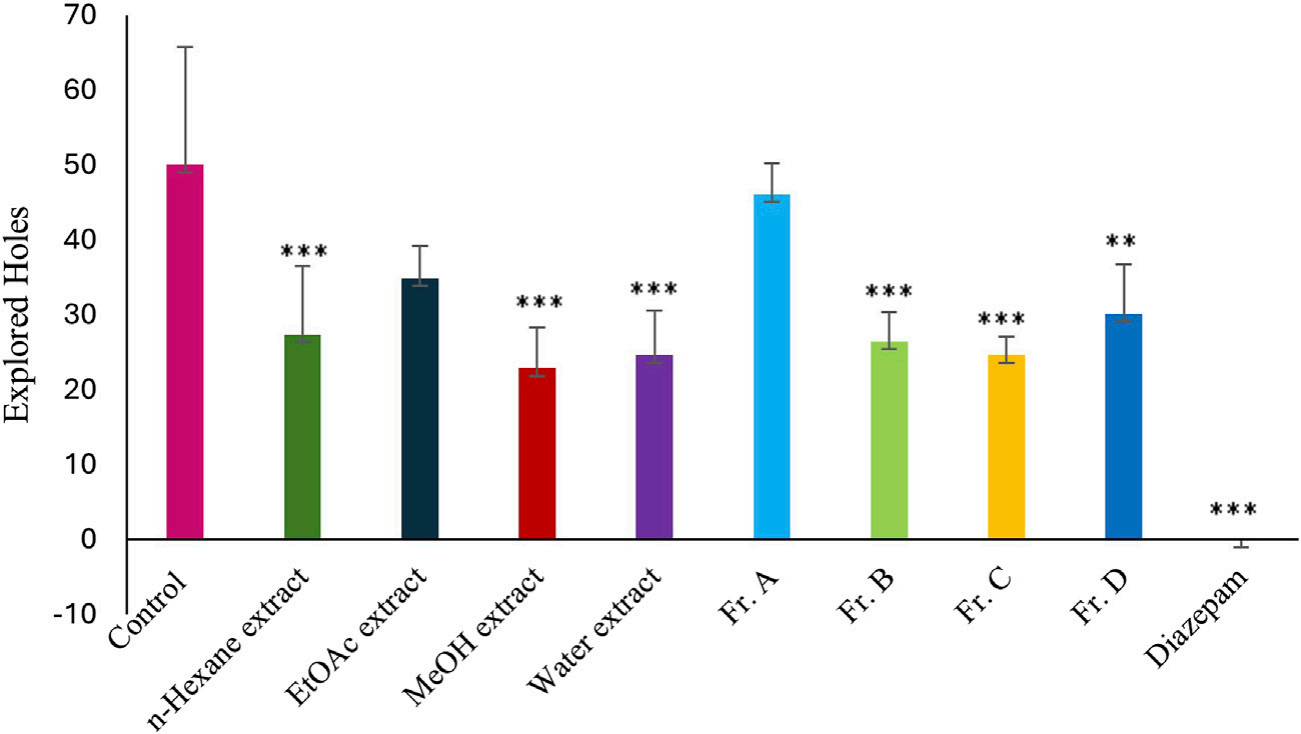
Effects of the test materials on hole-board test (*: *p* < 0,05; **: *p* < 0,01; ***: *p* < 0,001).

The powdered and dried aerial parts of *C. sicula* were extracted with *n*-hexane, ethyl acetate (EtOAc), and methanol (MeOH), respectively. In addition, water extract was prepared from dried plant. The yield of the extracts obtained was 0.63% for *n*-hexane, 1.42% for EtOAc, 22.97% for MeOH, and 20.94% for water extracts. The extracts, control and reference groups were evaluated according to re-establishment time of mice in the traction test and the number of explored holes in the hole-board test. The traction test findings demonstrate that EtOAc and MeOH extracts showed significant activity compared with the control group. When the hole-board test was evaluated, significant activity was observed in *n*-hexane, MeOH and water extracts compared to the control group. However, since the MeOH extract showed the highest activity in both experimental models, isolation studies were carried out on this extract. The successive bioassay-guided fractionation procedures were applied to the MeOH extract, and in this context Fr. A, Fr. B, Fr. C and Fr. D were tested with *in vivo* traction and hole-board tests. Fr. C showed significant activity in both experimental models. In addition, Fr. B and Fr. D showed mild activity in the traction test and significant activity in the hole board test (Table 1; Figures 1, 2).

**TABLE 1:** Sedative-anxiolytic effects of test materials determined by traction and hole-board tests.

Test material	Dose (mg/kg)	Traction test	Hole-board test
		Re-establishment Time (sec) ± S.E.M	Explored holes (during 5 min) ± S.E.M
Control	—	2.81 ± 1.66	50.01 ± 15.76
n-Hexane extract	100	3.91 ± 1.79	27.30 ± 9.20***
EtOAc extract	100	12.30 ± 3.65***	34.83 ± 4.40
MeOH extract	100	11.95 ± 5.21***	22.83 ± 5.49***
Water extract	100	4.99 ± 2.48	23.33 ± 6.56***
Fr. A	100	5.15 ± 2.06	46.05 ± 5.93
Fr. B	100	8.03 ± 1.92*	26.42 ± 3.93***
Fr. C	100	10.81 ± 1.78***	24.58 ± 4.12***
Fr. D	100	8.15 ± 0.12*	30.12 ± 2.46**
Diazepam	1	37.72 ± 6.05***	0.00 ± 0.00***

* : *p* < 0,05; **: *p* < 0,01; ***: *p* < 0,001; SEM:standard error of the mean.

**FIGURE 3:**
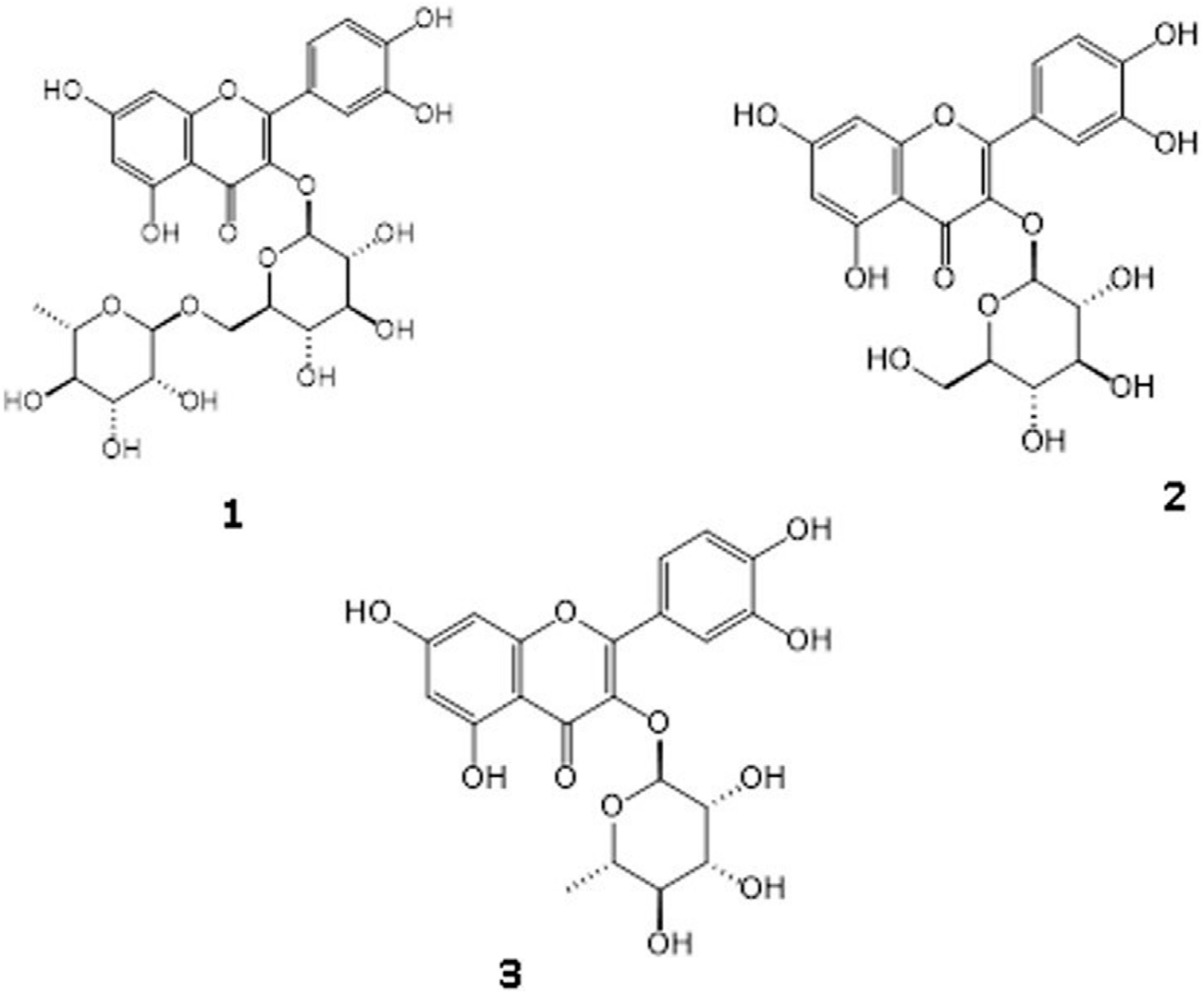
Identified three flavonoid glycosides as Rutin [1], Quercetin-3-O-glucoside [2], Quercetin 3-O-rhamnoside [3].

Column chromatography was carried out to the MeOH extract for isolation of possible active compounds, including polyamide, reversed-phase silica (RP-18) and Sephadex LH 20 techniques. Three flavonoid glycoside derivative compounds were isolated from Fr. C and Fr. D. The structures of these flavonoid glycosides were elucidated to be rutin, quercetin-3-*O*-glucoside, and quercetin-3-*O*-rhamnoside, by comparing the previous one- and two-dimensional NMR and ESIMS (-) datas (Figure 3) (Zhang et al., 2014; Gyeltshen et al., 2022).

**FIGURE 4:**
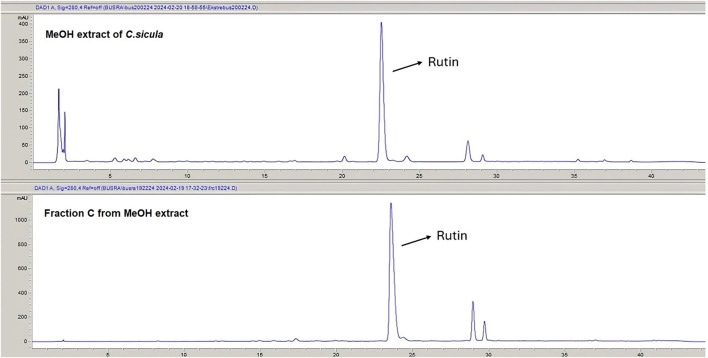
The HPLC chromatograms of the MeOH extract and fraction C.

Rutin content of the MeOH extract and fractions was quantitatively analyzed using the RP-HPLC-DAD method to identify the marker compound for the active extract. The rutin content of the MeOH extract of *C. sicula* was determined that 8 mg/100 mg dry extract. The rutin content of the fraction C was calculated as 76.7 mg/100 mg dry fraction. The HPLC chromatograms of the MeOH extract and the fraction C were given in Figure 4. Rutin was not to be found in fractions A, B and D. The retention time, test ranges, LOD, and LOQ values, in addition to the linear connection between peak area and concentration was given in Table 2.

**TABLE 2:** Retention times, linear relationships between peak areas and concentrations, test ranges, LOD and LOQ.

Compound	Retention time (min)	Standard curve	R2	Test range (µg/mL)	LOD (µg/mL)	LOQ (µg/mL)
Rutin	22.485	y = 18.31x + 165.51	0.999	1-1,000	0.064	0.193

y: peak area; x: concentration (mg/mL); LOD, limit of detection 3.3x SD/m; LOQ, limit of quantification 10xSD/m.

**FIGURE 5:**
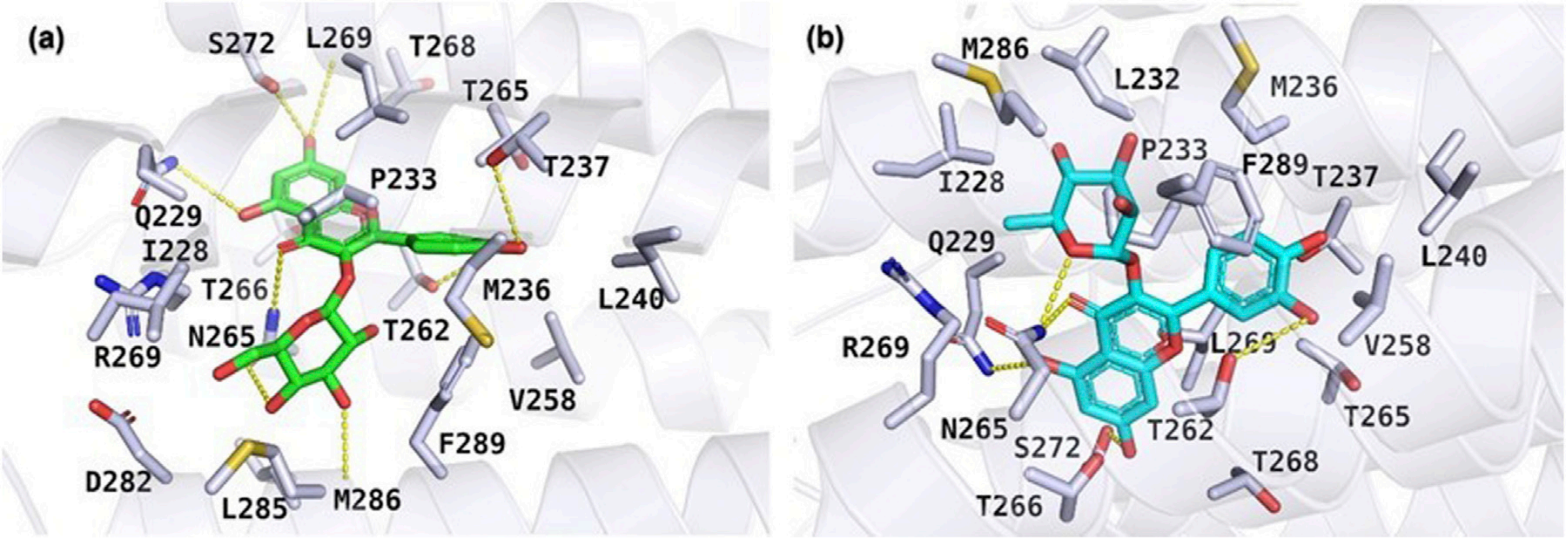
Molecular docking results illustrating the binding poses of **(A)** quercetin-3-O-glucoside and **(B)** quercetin-3-O-rhamnoside within the active site of the GABAA receptor (PDB ID: 6X3X). The docking poses were obtained using the Glide XP docking algorithm.

**FIGURE 6:**
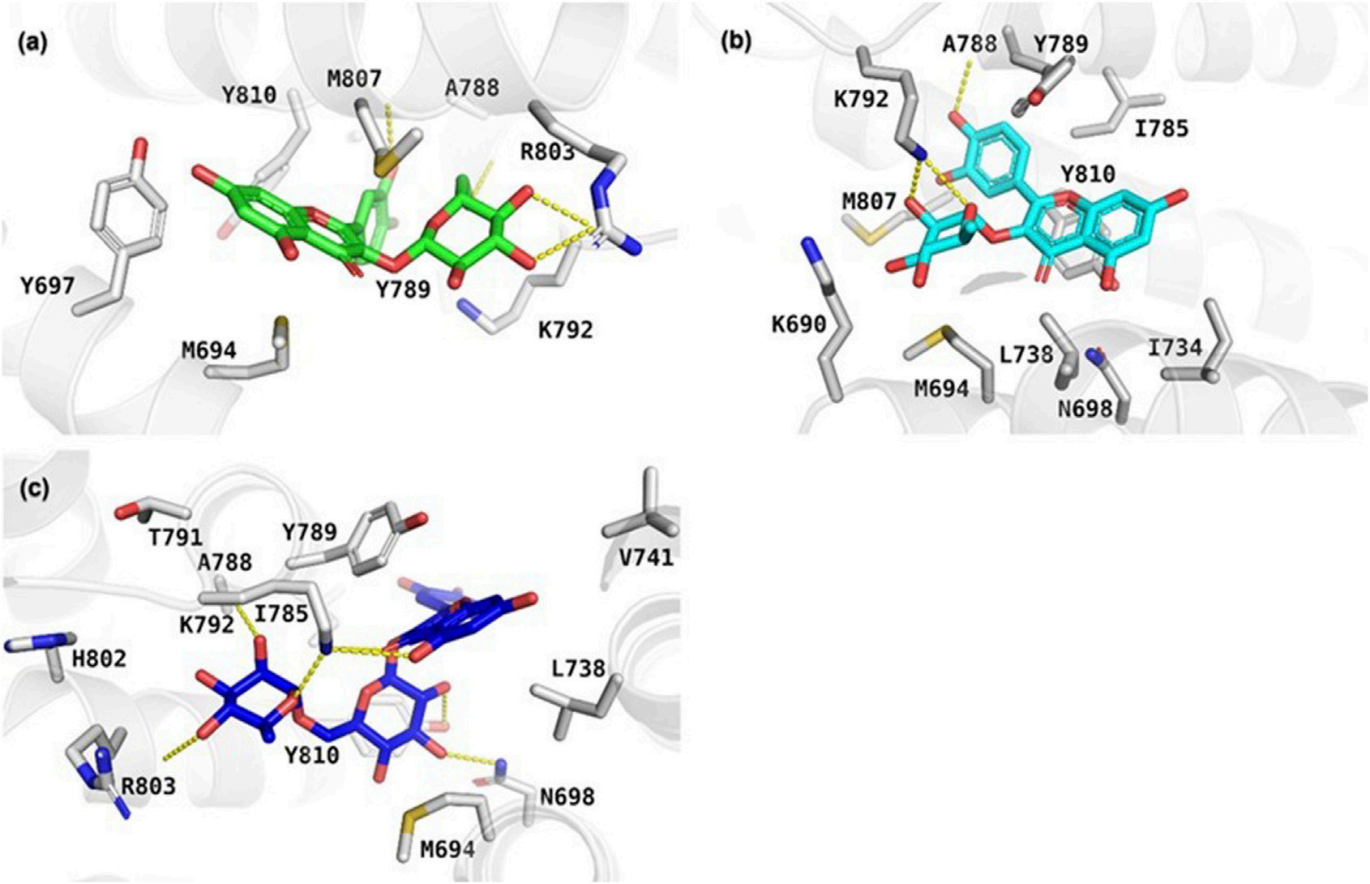
Depiction of the protein-ligand interaction patterns for **(A)** quercetin-3-O-glucoside, **(B)** quercetin-3-O-rhamnoside, and **(C)** rutin within the active pocket of the GABAB receptor (PDB ID: 6UO8).

The results of the molecular docking study include the interaction energies and protein-ligand interaction details of the anxiety-related receptors GABAA (PDB ID: 6X3X) and GABAB (PDB ID: 6UO8). This study investigated the compounds quercetin-3-O-glucoside, quercetin-3-O-rhamnoside, and rutin, as well as the cocrystal ligands diazepam and GS39783, respectively, in their interactions with the target protein structures. For the GABAA receptor, quercetin-3-*O*-glucoside demonstrated the highest docking score (−10.553) and formed hydrogen bonds with Asn265 (2.22 Å) and π-π stacking with Phe289 (4.75 Å). This strong binding affinity suggests that quercetin-3-O-glucoside could effectively interact with the GABAA receptor, potentially modulating its activity. Quercetin-3-*O*-rhamnoside showed good binding interactions with the GABAA receptor, with a docking score of −9.852. Its binding was primarily facilitated through hydrophobic interactions involving residues such as Val258, Leu259, Thr262, and several others (Figure 5). This indicates a stable interaction that might influence receptor function. Diazepam, used as a cocrystal ligand, displayed a docking score of −7.360 and interacted with specific residues in the receptor, including Met261, Thr262, and Asn265, which supports its known efficacy as an anxiolytic drug. Interestingly, rutin did not exhibit significant binding interactions with the GABAA receptor, as evidenced by the lack of a meaningful docking score. In the case of the GABAB receptor, quercetin-3-O-glucoside exhibited a docking score of −5.638 and formed hydrogen bonds with Ala788 (1.96 Å), Lys792 (1.83 and 2.55 Å), and Gly806 (2.00 Å). These interactions indicate a moderate binding affinity, suggesting quercetin-3-O-glucoside might influence GABAB receptor activity. Quercetin-3-O-rhamnoside, with a docking score of −4.804, and rutin, with a docking score of −6.135, also showed interactions with the GABAB receptor. Quercetin-3-*O*-rhamnoside formed hydrogen bonds with Ala788 (1.9 Å) and Lys792 (2.10 Å), while rutin formed hydrogen bonds with Ala788 (1.90 Å), Lys792 (2.08 Å), and Arg803 (2.49 Å). The hydrophobic interactions for both compounds involved residues such as Ile785, Tyr789, and Met807, which could contribute to their binding stability (Figure 6). GS39783, a co-crystal ligand, showed interactions with specific residues in the receptor, including Asn698 (2.09 Å), indicating its role in modulating GABAB receptor function. Overall, the molecular docking results suggest that quercetin-3-O-glucoside has the highest binding affinity for the GABAA receptor among the compounds, with significant hydrogen bonding and π-π stacking interactions. Quercetin-3-*O*-rhamnoside and rutin also show promising interactions, particularly with the GABAB receptor, highlighting their potential as modulators of these receptors. The detailed interaction energies (kcal/mol) and protein-ligand interaction details are provided in Table 3, which include specific amino acid residues involved in the binding and the nature of these interactions (hydrogen bonds, π-π stacking, and hydrophobic interactions).

**TABLE 3:** The interaction energies (kcal/mol) and protein-ligand interaction details of anxiety-related receptors GABAA (PDB ID: 6X3X) and GABAB (PDB ID: 6UO8) obtained from molecular docking study of compounds quercetin-3-O-glucoside, quercetin-3-O-rhamnoside, and rutin, and cocrystal ligands in target protein structure diazepam and GS39783, respectively. A and B in the table shows the chain names of GABAs.

Target proteins	Compounds	PubChem ID	Docking score	Protein-ligand interactions		
				H Bonds	π-π stacking	Hydrophobic
GABAA	Quercetin-3-O-glucoside	5280804	−10.553	A:Asn265 (2.22 Å)	A:Phe289 (4.75 Å)	A:Val258, A:Leu259, A:Thr262, A:Asn265, A:Thr266, A:Arg269, A:Glu270, A:Asp282, A:Leu285, A:Met286, A:Phe293, B:Ile228, B:Gln229, B:Leu232, B:Pro233, B:Met236, B:Thr237, B:Leu240, B:Thr265
	Quercetin-3-O-rhamnoside	5280459	−9.852	—	A:Phe289 (4.73 Å)	A:Val258, A:Leu259, A:Thr262, A:Asn265, A:Thr266, A:Arg269, A:Glu270, A:Met286, A:Phe293, B:Ile228, B:Gln229, B:Leu232, B:Pro233, B:Met236, B:Thr237, B:Leu240, B:Thr265, B:Thr268, B:Leu269, B:Ser272
	Diazepam	3016	−7.360	—	—	A:Met261, A:Thr262, A:Asn265, A:Arg269, A:Asp282, A:Leu285, A:Met286, A:Phe289, B:Ile228, B:Leu232, B:Pro233, B:Met236, B:Thr237, B:Thr265
GABAB	Quercetin-3-O-glucoside	5280804	−5.638	A:Ala788 (1.96 Å), A:Lys792 (1.83 and 2.55 Å), A:Gly806 (2.00 Å)		A:Ile785, A:Tyr798, A:Thr791, A:Ser793, A:His802, A:Arg803, A:Met807, A:Ile809, A:Tyr810, A:Asn811, B:Met694, B:Tyr697
	Quercetin-3-O-rhamnoside	5280459	−4.804	A:Ala788 (1.9 Å), A:Lys792 (2.10 Å)		A:Ile785, A:Tyr789, A:Arg803, A:Gly806, A:Met807, A:Tyr810, B:Lys690, B:Tyr691, B:Met694, B:Asn698, B:Ile734, B:Leu738
	Rutin	5280805	−6.135	A:Ala788 (1.90 Å), A:Lys792 (2.08 Å), A:Arg803 (2.49 Å), B:Asn698 (1.94 Å)		A:Ile785, A:Phe786, A:Tyr789, A:Thr791, A:His802, A:Gly806, A:Met807, A:Tyr810, B:Lys690, B:Tyr691, B:Met694, B:Tyr697,B:Ile734, B:Cys737, B:Leu738, B:Val741, B:Pro742
	GS39783	6604928	−4.928	B:Asn698 (2.09 Å)		A:Ile785, A:Ala788, A:Tyr789, A:Lys792, A:Gly806, A:Met807, A:Tyr810, A:Asn811, A:Val851, B:Met694, B:Tyr697, B:Leu738

## 4 Discussion

In this study, our team carried out a study to determine the sedative-anxiolytic activity of *C. sicula* plant, which is used for sedative-anxiolytic purposes in folk medicine, for the first time with *in vivo* experimental models. The hole-board test and the traction test were preferred as *in vivo* experimental models. The traction test is a commonly used approach for evaluating sedative and muscle-relaxant effects (Muhammad et al., 2013). In this study, the traction test was used to compare the sedative and muscle-relaxant properties of extracts derived from *C. sicula* with those of the reference drug, diazepam. The MeOH, Fr. C and the EtOAc extracts showed significant sedative and muscle relaxant activity compared with the control group (Table 1; Figure 1). The hole-board test is a recommended technique for examining any agent’s possible sedative and anxiolytic effects on mice. The head-dipping behavior is closely associated with a mice’s emotional condition (Takeda et al., 1998). Animal head dipping incidence is correlated with an increase in head dipping behavior (Viola et al., 1995). Table 1 indicates there was a statistically significant decrease in the number of head stings following the administration of *n*-hexane, MeOH, Fr. B, Fr. C, Fr. D, EtOAc extracts, and diazepam in the hole-board test compared to the control group (Figure 2). Benzodiazepine derivatives are drug groups that are mostly used in the treatment of anxiety disorders. Studies on benzodiazepines have shown that these drugs exert their anxiolytic effects through the GABAergic system by interacting with GABA receptors at neuronal synapses in the CNS (Katzung et al., 2018). This group of drugs causes an increase in membrane hyperpolarization by increasing the opening frequency of GABA-mediated chloride ion channels. In clinical applications, they are used in acute anxiety states, insomnia, skeletal muscle relaxation, and as an adjunct to anesthesia (Sarris et al., 2011). Consequently, in the present investigation, diazepam was used as the reference standard.

Numerous plants such as *Valeriana officinalis, Hypericum perforatum, Passiflora incarnata, Humulus lupulus, Lavandula angustifolia, Melissa officinalis, Piper methysticum, Tilia cordata, Matricaria chamomilla, Eschscholzia californica, Leonurus cardiaca* have been used traditionally to relieve the effects of anxiety (Werneke et al., 2004). It has been shown that people with anxiety often use natural resources and other complementary products in their treatment (Jäger and Saaby, 2011). Many of those plants are alkaloid-containing ones because alkaloids are known to interact strongly with CNS receptors. However, it has emerged in recent years that phenolics, especially flavonoids may also be involved in the brain’s receptor and enzyme systems, potentially having a variety of effects on the CNS (Girish et al., 2013). Flavonoids interact with a variety of receptors and signaling systems, including GABA receptors, to exhibit anxiolytic, sedative, anticonvulsant, and analgesic effects (Medina et al., 1997; Noguerón-Merino et al., 2015). Plant species that have a long history of usage as traditional folk medicines in Europe are among those where flavonoids are either shown to be active ingredients. For centuries chamomile (*Matricaria recutica*) has been used for its calming properties, which are attributed to apigenin (Viola et al., 1995). In traditional medicine, *Passiflora* species such *P. quadragularis* are used as sedatives and mild sedatives. Reports on the chemical composition of *P. quadragularis* leave extracts indicate the presence of flavonoids (Gazola et al., 2018). In the light of all these data, the flavonoid glycosides isolated from the methanol extract of *C. sicula*, whose sedative and anxiolytic activity was determined in *in vivo* experimental models, suggested that the activity may be mediated by these compounds. Therefore, the relationship of the three isolated flavonoid glycosides with GABAA and GABAB receptors was evaluated by *in silico* molecular docking study. These results of *in silico* molecular docking study provide detailed insights into the interaction energies and protein-ligand interaction patterns of the compounds quercetin-3-*O*-glucoside, quercetin-3-*O*-rhamnoside, and rutin with anxiety-related receptors. The docking scores indicate the strength of the binding interactions, while the specific residues involved in hydrogen bonding, π-π stacking, and hydrophobic contacts provide information on the molecular mechanisms of interaction. The data obtained from the *in silico* results of this study supported the results of the *in vivo* studies and suggested that the activity may be the result of the interaction of flavonoid glycosides interaction with GABA receptors. The increasing importance of such *in silico* studies lies in comprehending the pharmacological effects of natural compounds like flavonoid glycosides and exploring them as potential anxiolytic agents.

In the literature, the studies on flavonoids *in vivo* models have shown evidence of their impact on the CNS. Fernández et al., showed that the flavonoid glycosides linarin, 2S-hesperidin, 2S-neohesperidin, 2S-naringenin, diosmin, gossipyn, and rutin showed sedative anxiolytic activity with the hole-board, thiopental-induced sleep, and locomotor activity tests. And in the same study aglycone structures were found to be ineffective, sugar molecules contributed positively to sedative effect (Fernández et al., 2006). Chrysin, a flavone derived from *Passiflora coerulea* L. (Passifloraceae), had an anxiolytic effect by increasing the number of entry into and duration of time mice spent in the open arms in the elevated plus-maze test of anxiety. Chrysin also lengthened the duration of head dipping (Wolfman et al., 1994). In the study on *in vivo* analgesic, anti-inflammatory, sedative (open field model) and muscle relaxant (inclined plane and traction test) effects of a flavonoid derivative 3′4′78-tetrahydroxy-3-methoxyflavone isolated from *Pistacia chinensis* were evaluated. According to this study, it was reported that this compound showed significant sedative and muscle relaxant activity (Rauf et al., 2024) In the study on the sedative-anxiolytic effect of *Opuntia ficus-indica* (L.) Mill. fruits, the flavonoid derivatives isorhamnetin, isorhamnetin 3-O-glucoside, isorhamnetin 3-*O*-rutinoside, and kaempferol 3-*O*-rutinoside were isolated from the active fraction by bioactivity-directed fractionation. In this study bioassay systems; traction test, fireplace test, hole-board test, elevated plus-maze test, and open-field test were used for sedative and anxiolytic effect. As a result of this study, it was determined that this effect was mediated by flavonoids (Küpeli Akkol et al., 2020). Another study showed rutin and quercetin-3-*O*-glucoside were responsible for the anxiolytic and sedative-like effects of *Tilia americana* var. *mexicana* (Schltdl.) Hardin through the GABA and serotoninergic 5-HT1A receptors (Aguirre-Hernández et al., 2016).

The studies conducted *in silico* were used to clarify the anxiolytic-like mechanism of action of quercetin proven by *in vivo* studies. In a study, Islam et al., investigated the interaction of quercetin with GABA (α5), GABA (β1), and GABA (β2) receptors, and as a result, it was determined that the anxiolytic-like activity of this compound may be mediated by GABA receptors (Islam et al., 2022). In another study, 5-methoxyflavone was evaluated for sedative-hypnotic-like activity in mice and the mechanisms involved by molecular docking studies. This study indicated that 5-methoxyflavone, which is the flavonoid derivative, showed a good binding affinity to GABAA by H-bond interactions, justifying its sedative-hypnotic-like activity (Shanmugasundaram et al., 2018). Overall, this molecular docking study sheds light on the potential binding properties of the investigated compounds with anxiety-related receptors. Understanding these interactions contributes to the exploration of their potential anxiolytic effects and paves the way for future research in drug development and the utilization of natural compounds for anxiety treatment.

Studies on the sedative properties of *Capparis* species have been published in the literature. The sedative-hypnotic activity of aqueous extract, methanolic extract, methanolic fraction, and dichloromethane fractions was evaluated by open-field and pentobarbital-induced sleep tests. The results showed that the *Capparis spinosa* L. dichloromethane fraction has the highest effect, and the aqueous extract has the lowest effect compared to the other extracts and fractions (Khoramjouy et al., 2021). Another study investigates the antidepressant and anxiolytic activities of the methanol root extract of *Capparis thonningii* Schum. The extract showed anxiolytic-like effects on the elevated-plus maze test, hole-board test, and light-dark test (Ishola et al., 2015). This study is the first study evaluating the sedative and anxiolytic effects of *C. sicula*. The results verified traditional use.

Considering all the data and the literature, it becomes clear that the sedative-anxiolytic effect of *C. sicula*, a plant used for sedative purposes, may be carried on by the interaction of flavonoid glycosides with GABA receptors. Since each of these flavonoid glycosides exhibits various levels of activity, it may be preferable to use them in combinations or extracts rich in flavonoid concentration rather than as anxiolytics alone. However, further studies are needed to confirm the sedative and anxiolytic effects of these flavonoid glycosides and to elucidate their mechanisms of action.

## 5 Conclusion

In conclusion, the *in vivo* sedative-anxiolytic activity of *C. sicula*, which is traditionally used for sedative purposes, was evaluated for the first time. As this activity was observed in the phenolic-rich methanol extract, phenolics were focused on, the isolation of major substances that may be effective was carried out, and three flavonoid glycosides (1–3) were isolated. A quantification study was carried out on the major one of these isolated substances. The interactions of these three flavonoid glycosides with GABA receptors were proven by *in silico* studies. This research on phytochemistry, *in vivo*, and *in silico* has established the sedative-anxiolytic mechanism and validated the use of *C. sicula* for sedative purposes in traditional medicine. This research provides comprehensive information that can form a scientific basis for the use of *C. sicula* in the clinical management of sleep disorders and anxiety. Additionally, further investigations are required to clarify the function and mechanism of these flavonoid glycosides, the *in silico* activity of which was assessed.

## Data Availability

The original contributions presented in the study are included in the article/supplementary material, further inquiries can be directed to the corresponding authors.
